# The Retentive Strength of Laser-Sintered Cobalt-Chromium-Based Crowns after Pretreatment with a Desensitizing Paste Containing 8% Arginine and Calcium Carbonate

**DOI:** 10.3390/ijms19124082

**Published:** 2018-12-17

**Authors:** Raphael Pilo, Sharon Agar-Zoizner, Shaul Gelbard, Shifra Levartovsky

**Affiliations:** Department of Oral Rehabilitation, The Maurice and Gabriela Goldschleger School of Dental Medicine, Tel- Aviv University, Tel -Aviv 6997801, Israel; rafipilo@gmail.com (R.P.); sharon.agar@gmail.com (S.A.-Z.); s1gelbard@gmail.com (S.G.)

**Keywords:** desensitizing paste, dentin, retention, cements, cobalt-chromium

## Abstract

The retention of laser-sintered cobalt-chromium (Co-Cr)-based crowns were examined after dentin pretreatment with desensitizing paste containing 8% arginine and calcium carbonate (DP-ACC). Forty lower first molars were prepared using a standardized protocol. The Co-Cr crowns were produced using selective laser melting. The teeth were either pretreated with the desensitizing paste or not pretreated. After one week, each group was cemented with glass ionomer cement (GIC) or zinc phosphate cement (ZPC). Surface areas of the teeth were measured before cementation. After aging, a universal testing machine was used to test the retentive strength of the cemented crown-tooth assemblies. The debonded surfaces of the teeth and crowns were examined at 2.7× magnification. Pretreating the dentin surfaces with the desensitizing paste before cementation with GIC or ZPC did not affect the retention of the Co-Cr crowns. The retention of the GIC group (6.04 ± 1.10 MPa) was significantly higher than that of the ZPC group (2.75 ± 1.25 MPa). The predominant failure mode for the ZPC and the nontreated GIC group was adhesive cement-dentin failure; for the treated GIC group, it was adhesive cement-crown failure. The desensitizing paste can be safely used to reduce post-cementation sensitivity without reducing the retentive strength of Co-Cr crowns cemented with GIC or ZPC.

## 1. Introduction

Dentin hypersensitivity following tooth preparation and cementation of fixed partial dentures (FPDs) has been a common phenomenon [1]. Post-cementation complaints from patients are received for 20 to 30% of crowns inserted [2], and this rate remains at 6% and 3% after two and three years, respectively [3]. There are several explanations for this postoperative sensitivity. One outcome of aggressive tooth preparation is an increased number of opened and expanded dentinal tubules [4,5]. This condition is further aggravated by inadequate provisional restorations and removal of the smear layer due to acid etching induced by the cements [6]. Porcelain fused to metal (PFM) restorations, for example, are most commonly luted with zinc phosphate or glass ionomer cements (GICs), which are acidic in nature [7].

In an effort to control postoperative sensitivity, various desensitizing agents have been used to seal dentinal tubules before crown cementation; however, the literature is inconsistent regarding the effects of these agents on the retentive strength of FPDs. Sailer et al. [8,9] and Stawarczyk et al. [10,11] showed that glutaraldehyde/HEMA pretreatment and resin sealing of dentin following tooth preparation had a beneficial effect on the shear bond strength of self-adhesive resin cement. Other studies have reported that dentin desensitizing by means of glutaraldehyde-containing primers or dentin sealing by means of bonding agents did not affect the bond strength of the cements tested [12,13]. On the other hand, several studies have demonstrated that these agents decrease crown retention to some extent [14,15]. Aranha et al. [16] showed that specimens treated with dentin desensitizers (except Gluma) yielded significantly lower mean bond strengths than nontreated control specimens.

Recently, a new in-office Colgate Sensitive Pro-Relief Desensitizing Paste containing 8% arginine and calcium carbonate (DP-ACC) was shown to provide immediate and lasting relief from dentin hypersensitivity [17,18,19,20,21]. No significant difference in the bonding strength of composites to enamel or dentin pretreated with this desensitizing paste has been reported [22,23,24]. In addition, pretreating dentin surfaces with DP-ACC prior to cementation did not affect the retention of complete cast metal crowns luted with a glass ionomer cement (GIC) [25] or the retention of zirconium oxide crowns luted with a resin-modified GIC or a self-adhesive resin cement [26].

Recently, a new additive manufacturing technology operated by computer-aided design and computer-aided manufacturing (CAD/CAM), referred to as selective laser melting (SLM) technology, has been introduced for fabricating cobalt-chromium (Co-Cr) frameworks for PFM crowns. Co-Cr crowns produced with SLM exhibit a marginal and internal accuracy that is comparable to that of conventional production procedures but save time and facilitate laboratory procedures [27,28,29].

No information has been found in the literature concerning the influence of pretreating dentin with DP-ACC on the retentive strength of Co-Cr crowns produced by the SLM technology and cemented by zinc phosphate cement (ZPC) and glass ionomer cement (GIC), which are the most frequently used cements for luting metal-based restorations.

The aim of this in vitro study was to evaluate the effect of the pretreatment of dentin with DP-ACC on the retentive strength of SLM Co-Cr copings cemented by ZPC and GIC. The null hypotheses were as follows: (1) the retentive strength of the SLM Co-Cr copings cemented by ZPC and GIC to human extracted teeth is not affected by DP-ACC, and (2) the retentive strength of the two cements is similar.

## 2. Results

The retentive strength (mean, SD) of the treated and untreated cementation groups are presented in Table 1. Pretreating the dentin surfaces with DP-ACC prior to cementation with either GIC or ZPC did not affect the retentive strength of the Co-Cr copings (*p* = 0.780). The retention obtained with Fuji I capsules (GIC) was significantly (*p* = 0.001) higher than that obtained with Harvard Cement OptiCaps (ZPC). The interaction between cement and dentin treatment was not significant (*p* = 0.208).

**Figure 1:**
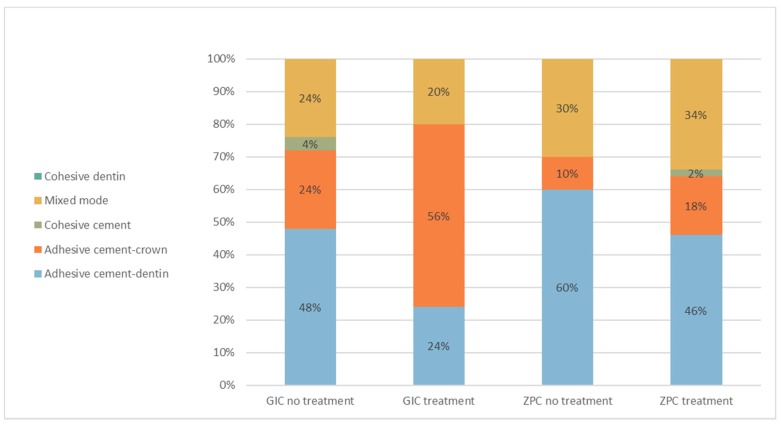
Distribution of failure modes (number of surfaces) for each cementation group.

Examination by magnifying glasses of the failure mode after the dislodgment of the crown revealed that for ZPC, the predominant failure mode was adhesive cement-dentin failure. In 85% of the surfaces, all (53%) or part (32%) of the surface of the crown was covered with cement, and the rest were detected on the dentin (Figure 1). This mode of failure was consistent regardless of whether the dentin was pretreated with DP-ACC or not. In the GIC group, the predominant failure mode was adhesive cement-crown failure. In 62% of the surfaces, all (40%) or part (22%) of the surface of the dentin was covered with cement, and the rest were detected on the crown (Figure 1). This failure mode was inconsistent between the groups. In the nontreated group, more surfaces exhibited the adhesive cement-dentin mode of failure; in the treated group, more surfaces exhibited the adhesive cement-crown mode of failure (Figure 1). Cohesive cement failure was barely seen in all groups, while cohesive dentin failure did not occur.

**Figure 2:**
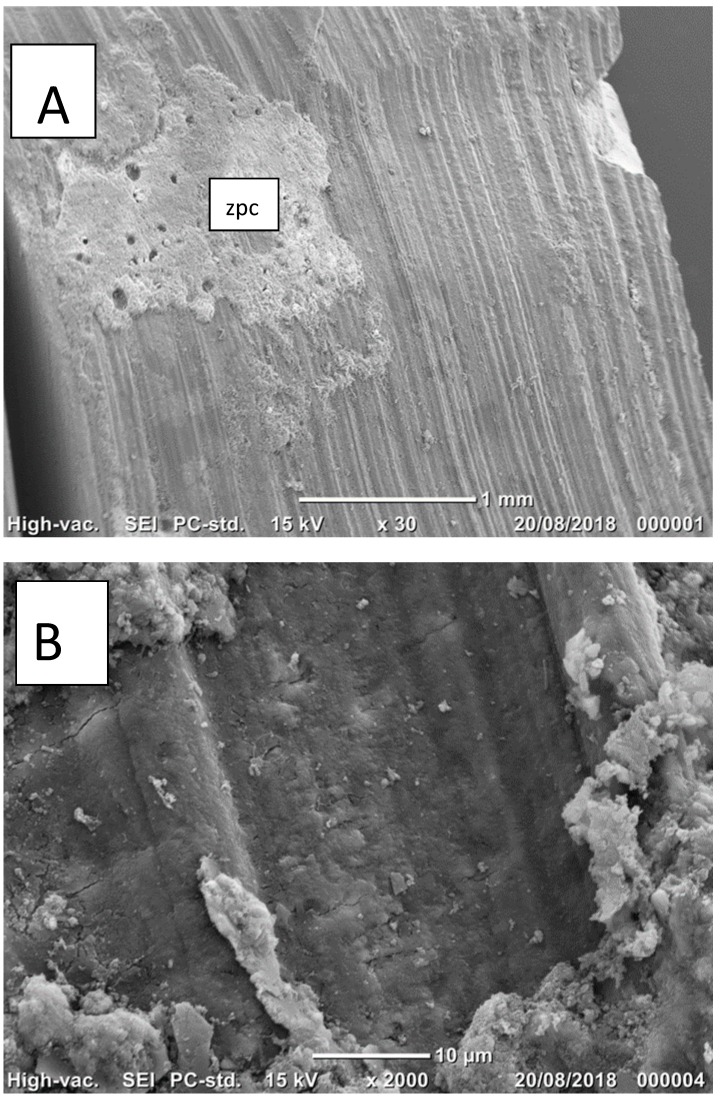

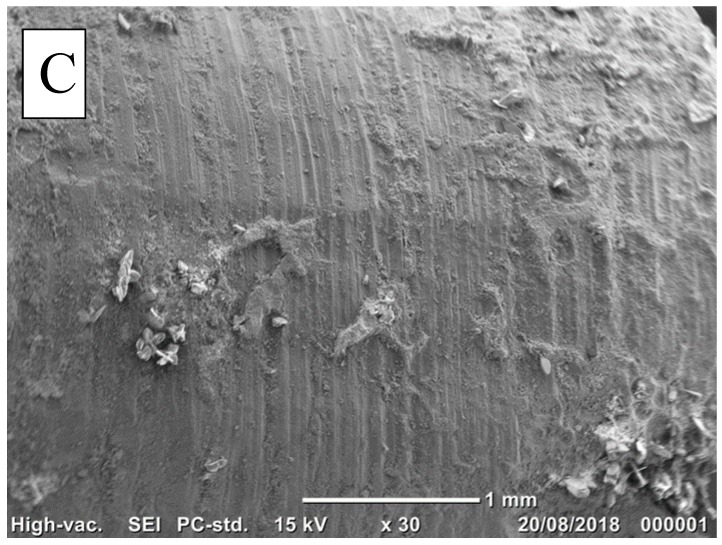
**A**–**C** (ZPC group). Scanning electron microscopy image of the untreated (**A**,**B**) and treated (**C**) dentin surfaces after failure illustrating the mixed mode of failure; the majority exhibited adhesive cement-dentin failure, while most of the dentin surface exhibited longitudinal striations of the bur, with only a small part covered with cement (ZPC). This type of predominantly adhesive cement-dentin failure was typical of the ZPC group.

**Figure 3:**
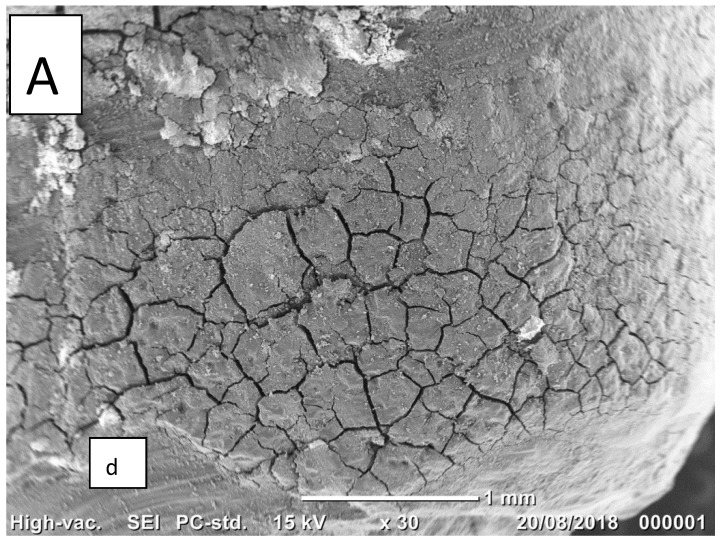

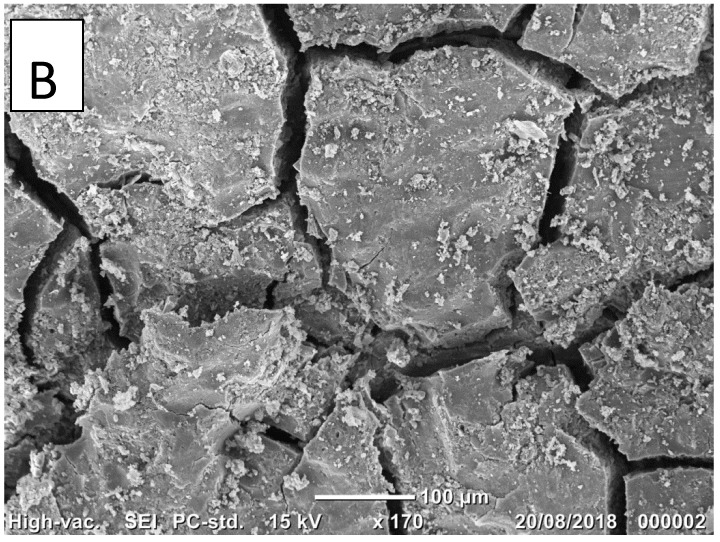
**A**,**B** (GIC group). Scanning electron microscopy images of the treated dentin surface after failure. (**A**) Most of the dentin surface is covered with cement (30×), and only a small part of the dentin is exposed (d), demonstrating the striations of the bur. (**B**) At greater magnification (170×), craze lines in the cement layer caused by the dehydration process are evident.

Scanning electron microscopy (SEM) images of the dentin surfaces illustrating the various modes of failure are presented for the ZPC (Figure 2a–c) and GIC (Figure 3a,b) groups. Figure 2a–c illustrates the mixed mode of failure, whereas most of the dentin surface exhibits longitudinal striations of the bur, with a small part covered with the ZPC. This type of failure was consistent in the untreated (A,B), as well as the treated (C) ZPC groups.

Figure 3a,b illustrates the adhesive crown cement mode of failure, with most of the dentin surface covered with cement. This mode of failure was typical of the treated GIC group.

## 3. Discussion

Colgate Sensitive Pro-Relief Desensitizing Paste contains arginine, bicarbonate, and calcium carbonate and is highly effective in occluding dentin tubules, as was previously demonstrated by confocal laser scanning microscopy (CLSM) and SEM [30]. This paste has been shown to physically plug and seal exposed dentin tubules and to effectively provide dentin hypersensitivity relief [31], which has been reported to last for up to 28 days and to reduce the post-preparation and post-cementation sensitivity of vital teeth that serve as abutments for FPDs; however, this treatment may be advocated only if retentive strength is not affected [20]. 

In the current study, the retention of cobalt-chromium-based (Co-Cr) copings was tested one week after dentin pretreatment with DP-ACC in order to resemble a period of reevaluation prior to final cementation. The luting agents used in this study were GIC and ZPC. Both cements are acid-base materials associated with post-cementation sensitivity, which can last for a week [32]. GIC and ZPC are popular choices for luting metal-based restorations. GIC relies on both the mechanical retention to surface irregularities and the chelation to calcium in the tooth structure, while ZPC relies only on mechanical retention to both dentin and crown surfaces [33,34]. GICs are naturally adhesive to dentin; initially due to the presence of polyacrylic acid, forming hydrogen bonds between the free carboxyl groups and the strongly bound water layers residing on the surface of the dentin. Subsequently they are gradually replaced by ionic bonds involving mainly calcium in the mineral phase at the surface of the dentin and carboxylate groups in the cement. Imaging of dentin surfaces treated with DP-ACC reveals surface coverage including the tubule orifices. This surface coverage may thus interfere with the interaction of the cement with the mineral phase of the surface dentin as well as block the longitudinal striations of the bur and, thus, impair both the micromechanical and chemical mode of action of the cements, affecting the bond strength/retention. 

The current results support the first null hypothesis of the study, implying that pretreatment with DP-ACC would have no effect on the retentive strength of SLM Co-Cr crown copings cemented to human extracted teeth with GIC or ZPC. Our results are in agreement with those of another study that demonstrated that pretreating dentin surfaces with DP-ACC prior to cementation did not affect the retention of complete cast metal crowns luted with GIC [25]. Moreover, in the aforementioned study, pretreatment with DP-ACC showed the best retention of complete cast metal crowns compared to all other dentin desensitizers tested. Pilo et al. [26] showed the same results with zirconium oxide (Y-TZP) crowns luted with either resin-modified glass ionomer cement (RMGIC) or self-adhesive resin cement (SARC).

Our second null hypothesis was rejected because the retentive strength of GIC was significantly higher than that of ZPC. This conclusion is in accordance with a previous study of Wiskott et al. [35] demonstrating that crowns luted with resin composite cement and GIC were more resistant to dynamic lateral loading than those luted using ZPC. On the other hand, Gorodovsky et al. [36] reported no significant difference between the retention of zinc phosphate and that of glass ionomer. A review of the research of different cements did not reveal a consistent conclusion about the retentive strength of GIC in comparison with that of ZPC. Some studies showed a higher retentive strength for ZPC, and others reported a higher retentive strength for GIC; some showed no significant difference between the two cements [33]. However, it should be noted that all the aforementioned ZPC studies used the classic Harvard Cement normal setting, whereas in the current study, the newer Harvard Cement OptiCaps was used. The latter is the capsulated version intended to overcome mistakes in mixing and dosing. It has been shown that Vickers hardness increases with more powder with a rise in mean values following an exponential curve ranging between 34 and 66 MPa [37]. Comparison studies between hand-mixed and capsulated ZPC have not been reported yet.

The lack of effect on the retentive strength of ZPC from DP-ACC was also verified by an absence of change in failure mode, which was mainly adhesive cement-dentin failure. This finding implies that all or most of the crown was covered by ZPC, probably due to surface irregularities of the intaglio of the Co-Cr copings. In the GIC group, the predominant failure mode was adhesive cement-crown failure. This finding implies that all or most of the dentin was covered by GIC, probably due to the chemical interaction between the calcium in the mineral phase at the surface of the dentin and carboxylate groups in the cement, implying that in spite of the surface coverage by the DP-ACC, many calcium ions are still available for bonding. Although the DP-ACC did not affect the retentive strength, the failure mode varied between the groups; in the nontreated group, most of the cement remained on the crown, while in the treated group, most of the dentin was covered with cement. These differences might be explained by chelation between the polyalkenoic chains in GIC also with the calcium carbonate contained in the DP-ACC, which physically plugs and seals exposed dentin tubules.

This is an in vitro study, hence, the subjective desensitizing effect of the DP-ACC on vital teeth prepared for FPD must be validated in clinical studies before being recommended for use.

## 4. Materials and Methods

The study sample comprised forty freshly extracted, caries-free, intact lower first molars that were extracted for periodontal reasons (age range 40–60). Approval from the Ethics Committee of Tel Aviv University was obtained (#21-08-16), and all individuals signed an informed consent.

The teeth were stored in a germ-free 0.1% thymol tap water solution at room temperature for a maximum of two weeks until experimentation. Each tooth was suspended in the middle of an aluminum ring and was mounted 2 mm apical to the cementoenamel junction (CEJ) in poly(methyl methacrylate) resin (Quick resin, Ivoclar, Schaan, Liechtenstein) after notching the roots for retention purposes. The mounted teeth were stored in tap water at room temperature at all times.

A standardized protocol yielding an axial height of 5 mm and a 10° taper was followed for preparation. The occlusal surface was sectioned perpendicular to the long axis with a water-cooled precision saw (Isomet Plus, Buehler, IL, USA). A 0.4-mm, 360° chamfer finish line located 1 mm above the CEJ with a 10° taper preparation was obtained by a rigidly secured, high-speed handpiece equipped with a diamond bur (C1-Strauss, Ra’anana, Israel) mounted on a custom-designed, surveyor-like apparatus. A new diamond bur was used for each tooth.

The prepared teeth were digitally scanned by a laboratory scanner (Series 7, Dental Wing, Letourneux, Montreal, Canada) operated by blue light and equipped with five axes of rotation, and STL files were obtained. Forty Co-Cr copings were produced using an SLM system (Eosint M 280, EOS, Krailling, Germany) at a commercial dental laboratory (MS Systems, Or Yehuda, Israel). The CAD-CAM Co-Cr cores were 1.0 mm thick with a 50 µm virtual cement spacer layer. To facilitate tensile loading, an occlusal loop (4-mm outer diameter and 2-mm inner diameter) was designed extending coronally from the occlusal surface [38]. The teeth were randomly assigned to two groups (2 × 20). In one group, the dentin surfaces were pretreated with DP-ACC using prophy cups under light pressure according to the manufacturer’s recommendations. In the second group (the control group), the dentin surfaces were not pretreated. After placing the Co-Cr copings on each tooth, they were stored at 37 °C under 100% humidity for one week, resembling a period of reevaluation prior to final cementation. Two luting cements were evaluated (2 × 10) in each group: a GIC (GC Fuji I Capsule, GC, Tokyo, Japan) and a ZPC (Harvard Cem OptiCaps, Harvard Dental International, GmbH). The areas of the axial and occlusal surfaces of each prepared tooth were measured prior to cementation, as previously described [38]. The cements were used according to the manufacturer’s recommendations. Cementing each crown to its tooth was conducted in a standardized manner under a constant load of 50 N (Force gauge, FG 20, Lutron, Taiwan) for 10 min and then allowed to set for 24 h.

The cemented crown-tooth assemblies were stored in tap water at 37 °C for two weeks, followed by thermal cycling between water temperatures of 5 and 55 °C for 5000 cycles with a 10 s dwell time (Y. Manes, Tel-Aviv, Israel). After thermal cycling, the crown-tooth assemblies were subjected to dislodgment forces through a 1.2-mm diameter metal cable entangled through the occlusal loop along the apico-occlusal axis using a universal testing machine (Instron, Model 4502, Instron Corp., Buckinghamshire, UK) at a crosshead speed of 1 mm/min until failure. The force at dislodgment was recorded and divided by the total surface area of each prepared sample to yield the retention value (Pa).

The debonded surfaces of the teeth and crowns were examined with magnifying glasses at 2.7× magnification (Orascoptic, Middleton, WI, USA). Each surface of the dentin-crown interface was analyzed separately (five surfaces per tooth). Failure was classified based on the criteria presented in Table 2.

A separate analysis was performed for each matched Co-Cr tooth surface (buccal, lingual, mesial, distal, and occlusal). For each category, the number of surfaces was counted and presented as a percentage of all the surfaces for the specific cement.

To analyze the dentin surfaces, the debonded surfaces of some teeth representing different failure categories from each group were examined under an SEM (Quantum 2000) in high vacuum mode following gold sputter-coating. The acquisition conditions were as follows: 25 kV, 90 µA and 40–1000× magnification.

### Statistical Analysis

Retentive strength was evaluated using two-way analysis of variance (ANOVA) with repeated measures; cement (*n* = 2) and pretreatment (*n* = 2) were the independent variables. The level of significance was 0.05.

## 5. Conclusions

An 8.0% arginine and calcium carbonate in-house desensitizing paste can be safely used on dentin to reduce post-cementation sensitivity without compromising the retention of SLM Co-Cr crowns cemented with either ZPC or GIC.

## Figures and Tables

**Table 1:** Mean (SD) retentive strength (MPa) of the cobalt-chromium-based crown for all cementation groups.

Cement Type	Treatment	Sample No.	Mean Retentive Value (MPa)	Standard Deviation
GIC	1	10	6.39	1.06
	2	10	5.73	1.10
	Total	20	6.04	1.10
ZPC	1	10	2.39	0.99
	2	10	3.10	1.44
	Total	20	2.75	1.25
Total	1	20	4.29	2.27
	2	20	4.41	1.83
	Total	40	4.36	2.03

Treatment: 1, Without pretreatment with DP-ACC (control); 2, With pretreatment with DP-ACC.

**Table 2:** Classification of failure criteria.

Classification	Description	Criteria
1	Cement principally on crown surface	Adhesive cement-dentin
2	Cement principally on dentin surface	Adhesive cement-crown
3	Cement equally distributed on dentin & crown surfaces	Cohesive cement
4	Mixed mode	Adhesive & cohesive cement
5	Fracture of the tooth	Cohesive dentin
